# THE EFFECTS OF ENVIRONMENT-INDUCED RELAXATION ON COGNITIVE FORAGING UNDER STRESS IN MIDLIFE AND BEYOND

**DOI:** 10.1093/geroni/igac059.2947

**Published:** 2022-12-20

**Authors:** Jessie Chin, Shannon Mejía, Smit Desai, Faith-Christina Washington, Durga Ramesh, Falguni Deshpande, Sanjana Dash

**Affiliations:** University of Illinois at Urbana-Champaign, Champaign, Illinois, United States; University of Illinois at Urbana-Champaign, Champaign, Illinois, United States; University of Illinois at Urbana-Champaign, Champaign, Illinois, United States; University of Illinois at Urbana-Champaign, Champaign, Illinois, United States; University of Illinois at Urbana-Champaign, Champaign, Illinois, United States; University of Illinois at Urbana-Champaign, Champaign, Illinois, United States; University of Illinois at Urbana-Champaign, Champaign, Illinois, United States

## Abstract

We defined enrichment seeking as the capacity for adults to engage in the novel and intellectually challenging activities for exploring diverse experience. Although enrichment seeking is associated with cognitive resilience in older adulthood, the tendency for older adults to adopt explorative behavior decreases with age in concert with decline in executive control. The goal of our study was to examine to what extent older adults can adjust their cognitive foraging performance under stress by inducing relaxation through the task environment. We used cognitive foraging games (including word search puzzle games) to understand how older adults optimize their search performance by the tradeoff between exploration (seeking new information) and exploitation (staying at old information). A modified Trier paradigm, where game performance was observed by a neurologist via video conferencing, induced stress; relaxation was manipulated by turning on an electric fireplace in the study room. The study followed a 2 (relaxation) X 2 (stress) within-subjects experimental design. Sixty-one adults (34 middle-age; 27 older adults; 54% women) played games on a tablet under stress / no stress conditions with and without the fireplace in a simulated smart home environment. The interaction effect of relaxation and stress on cognitive foraging performance was significant, indicating that with the relaxation elements in the environment (i.e., a fireplace), older adults are likely to have better cognitive foraging performance under stress. The association can be in part explained by individual differences in cognitive abilities and strategies. Implications on facilitating enrichment seeking through environmental factors are discussed.

